# EMBuilder: A Template Matching-based Automatic Model-building Program for High-resolution Cryo-Electron Microscopy Maps

**DOI:** 10.1038/s41598-017-02725-w

**Published:** 2017-06-01

**Authors:** Niyun Zhou, Hongwei Wang, Jiawei Wang

**Affiliations:** 1 MOE Key Laboratory of Protein Science, Tsinghua University, Beijing, 100084 China; 2 School of Life Sciences, Tsinghua University, Beijing, 100084 China; 3 State Key Laboratory of Membrane Biology, Tsinghua University, Beijing, 100084 China

## Abstract

The resolution of electron-potential maps in single-particle cryo-electron microscopy (cryoEM) is approaching atomic or near- atomic resolution. However, no program currently exists for *de novo* cryoEM model building at resolutions exceeding beyond 3.5 Å. Here, we present a program, EMBuilder, based on template matching, to generate cryoEM models at high resolution. The program identifies features in both secondary-structure and Cα stages. In the secondary structure stage, helices and strands are identified with pre-computed templates, and the voxel size of the entire map is then refined to account for microscopic magnification errors. The identified secondary structures are then extended from both ends in the Cα stage via a log-likelihood (LLK) target function, and if possible, the side chains are also assigned. This program can build models of large proteins (~1 MDa) in a reasonable amount of time (~1 day) and thus has the potential to greatly decrease the manual workload required for model building of high-resolution cryoEM maps.

## Introduction

In recent years, substantial progress has been made in single-particle analysis (SPA) and has led to a resolution revolution in cryo-electron microscopy (cryoEM)^1^. The resolution of cryoEM structures is improving because of advancements in both hardware and software. An increasing number of structures of small proteins with low symmetry have achieved resolutions of ~3.5 Å^2^ and even ~2 Å^3^ or better. Interpreting these cryoEM maps as atomic models manually would be a difficult and tedious task. Therefore, fully automatic model-building programs for cryoEM maps are urgently needed.

Previous cryoEM model-building programs have mainly focused on medium-resolution (3.5–6 Å)^4^ maps. The information content in a medium-resolution cryoEM map is insufficient to precisely interpret the main chain of a protein. Accordingly, most of the programs have added various types of external information (*i.e*., structure-prediction-related information) to complement model building. A *de novo* model-building method^5^ based on ROSETTA^6^ combines the sequence-derived prediction of backbone conformations and side chain information with a cryoEM potential map to build a backbone and assign sequences. Gorgon^7^ is an interactive modeling toolkit that performs *de novo* model building for cryoEM maps at resolutions of 3.5 to 10 Å, which combines sequence-based secondary structure prediction with feature detection and geometric modeling techniques to trace the backbones in cryoEM maps. EM-fold^8^ is a method that combines secondary structure prediction with a Monte Carlo assembly algorithm and Rosetta refinement procedures to build topology models for medium-resolution cryoEM maps. Pathwalking^9^ is based on the Traveling Salesman Problem (TSP) and traces the backbones in cryoEM maps. This method places the pseudo-atoms and traces the backbone by solving TSP on the basis of the placed pseudo-atoms. The secondary structure elements (SSEs) in the backbone are thus identified and corrected.

However, because of the resolution revolution of cryoEM maps (*i.e*., beyond 3.5 Å), the information content in the map itself is now adequate enough to trace the main chain and part of the side chain. Therefore, the map can be directly interpreted by matching it with pre-built models or templates. In X-ray crystallography, numerous automatic model-building programs have been developed by using the template-matching method^10^. Buccaneer^11^, the successor of FFFear^12^, uses an oriented electron-density likelihood target function to identify likely Cα positions and then expands these Cαs to the extended main chain fragments. Side chain assignment is performed by applying the electron-density likelihood target function after the main chain extension. RESOLVE^13^ identifies secondary structures and extends them with pre-built segment libraries constructed from the Protein Data Bank (PDB) database. C-Alpha Pattern Recognition Algorithm (CAPRA)^14^ uses pattern-recognition techniques and a neural network to predict the candidate atoms closest to true Cα atoms. ARP/wARP^15^ is a software suite for model building, refinement and validation. It uses density recognition-driven procedures to place and remove atoms, thereby limiting the resolution of the data to 2.5 Å or higher. Automatic Crystallographic Map Interpreter (ACMI)^16^ uses probabilistic inference to predict the backbone layout and a statistical sampling method to produce an accurate and physically feasible set of structures and side chain templates to sample side chains. All of these programs were developed to address the problem of model building specific in crystallography. For example, the templates used in RESOLVE are derived from X-ray crystal structures and hence are suboptimal templates for cryoEM maps. Meanwhile the refinement procedure improves the phases of crystallographic maps iteratively, but no phase problem exists in cryoEM maps. Therefore, these distinct characteristics of cryoEM maps should stimulate the development of novel model-building programs for high-resolution cryoEM maps.

The most important problem that must be overcome in model building of high-resolution cryoEM maps is that the voxel size may be imprecise because of microscopic magnification errors that occur during data collection^17^. Hence, the voxel size should be corrected before model building to prevent errors in the final model. Furthermore, the noise level and scattering factors of cryoEM maps differ from that of crystallographic maps. Thus, the templates derived from crystallographic maps are not suitable for cryoEM maps, and it is crucial to re-design the algorithms and re-tune the parameters for cryoEM map applications. To this end, we present a dedicated program—EMBuilder—based on the template-matching method for model building specifically designed for high-resolution cryoEM maps.

## Methods

### Program Workflow

**Figure 1:**
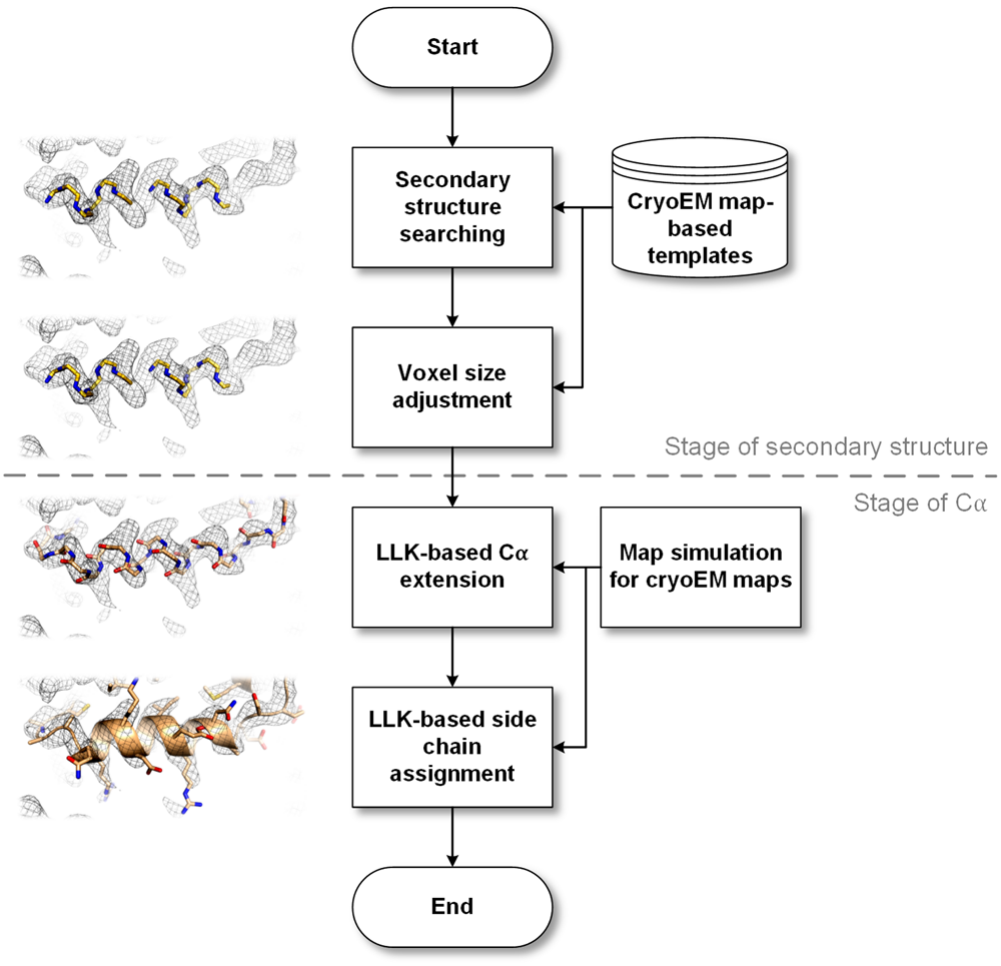
Workflow of EMBuilder. First, the program works on the secondary structure stage. Secondary structure templates are searched and placed in the map. Then, voxel size refinement is performed on these templates. Subsequently, the program works on the Cα stage. The reference map is adjusted to the same scale as the working map. The Cα target and side chain target functions are created on the basis of the scaled reference map, and then, Cα extension and side chain assignment are performed.

EMBuilder is based on the template-matching method and processes cryoEM maps through two stages: secondary-structure stage and Cα stage (Fig. 1). In the first stage, the positions of secondary structures are searched in the working map (input map) on the basis of the templates, including α-helix and β-stand, generated from previously known cryoEM structures. These potential secondary structure positions and voxel size are then refined using the correlation coefficient (CC) as the refinement target function. Once the voxel size is corrected, the program proceeds to the Cα stage. Another cryoEM map which is used as a reference is adjusted to the same scale as the working map by using a map simulation procedure. The scaled reference map, along with a known atomic model, is used to build Cα and side chain log-likelihood (LLK) target function. Then, candidate Cαs are extended from both ends of the secondary structures to complete the main chain model based on the Cα LLK target function learned from the scaled reference map. As an option, a side chain is assigned if a sequence of the working map is available. These LLK related procedures are similar to that in Buccaneer^11^. Clipper^18^ and CCP4 coordinate libraries^19^ are used to handle ccp4/mrc-format maps and PDB files. The individual functionalities are discussed below in details.

### Templates from CryoEM Map

We generated a helix template that was 6 residues long and a strand template that was 4 residues long. Each template consisted of a PDB coordinate set, a mean map and a correlation map. These two templates were used for the subsequent secondary structure identification and voxel size refinement.

The calculation of the mean map and the correlation map of the template was similar to that used in RESOLVE^20^. A helix (133–138) in myoglobin (PDB entry 1a6m)^21, 22^ and a strand (105–108) in carboxypeptidase A (PDB entry 1bav)^21, 23^ were chosen as standard helix and strand respectively. The cryoEM map of glutamate dehydrogenase (GDH) (PDB entry 5a1a, EMDB entry 2984)^21, 24, 25^ was low-pass filtered to 2.5 Å, 3.0 Å and 3.5 Å. All helices/strands of GDH were rotated and translated onto the above standard helix/strand, and only helices/strands with root-mean-square deviations (RMSDs) less than 0.5 were selected for further analysis. The cryoEM grid points of each selected helix/strand within 15 Å of the center of mass (COM) of the standard helix/strand were used for third-order interpolation on a standard grid (expressed as *S*_*nk*_, where *n* represents the selected helices/strands, and *k* represents the interpolated grid points in each helix/strand). The mean map *S* ^*mean*^ was calculated by separately averaging grid points from all of the selected helices/strands of GDH:

1 $${{S}}_{{k}}^{{mean}}=\frac{1}{{n}}\sum _{n}{{S}}_{{nk}}$$

The correlation map was calculated by averaging the CCs between the above-mentioned mean map and individual cryoEM maps of helices/strands. For each *S*_*nk*_, the distance between *S*_*nk*_ and the nearest atom in the standard helix/strand models was calculated (expressed as *L*_*nk*_). All *L*_*nk*_ <15 Å were divided into 30 groups with 0.5 Å intervals, expressed as *G*_*i*_ (where *i* represents the group number, *i*∈ {1, 2, …, 29, 30}). For each group *G*_*i*_, the CCs between all *S*_*nk*_ within the same *G*_*i*_ and ${{S}}_{{k}}^{{mean}}$ were calculated and averaged (expressed as *CC*_*i*_):

2 $${C}{{C}}_{{i}}=\frac{1}{{n}}\sum _{n}{{\rm{\rho }}}_{n}({S}_{nk},{S}_{k}^{mean}),k\in {G}_{i},i\in \{1,2,\mathrm{...},29,30\}$$

The value of each grid point of correlation map ${{S}}_{{k}}^{{corr}}$ was assigned as the *CC*_*i*_ where the group *i* was the grid point belongs to.

### Secondary Structure Searching and Voxel Size Refinement

The refinement of the voxel size is typically performed by comparing the cryoEM map with a corresponding atomic model and adjusting the voxel size until the CC between the map and model is maximized^26^. However, no atomic model currently exists for such analysis before model building. In our method, we performed a 7-dimensional refinement (3D rotation, 3D transition and voxel size) on helix/strand templates to correct the voxel size of the maps.

The initial position of the helix/strand was roughly determined through a fast searching method, as used in Coot^27^, to save the computation time compared with the CC-based searching method. It only uses several values of grid points of the map to determine the potential positions of helix/strand, *e.g*. the grid values at the Cα positions of the template. Therefore, it is very fast but quite imprecise. Once the rough positions are determined, the helix/strand template maps were then placed on these positions. The CC between the working map and the mean map of the placed helix/strand template was calculated and weighted by the corresponding correlation map. The top 20 helices/strands with CCs greater than 0.3 were selected (expressed as *S*_*n*_, where *n* represents the number of helices/strands, *n*∈ {1, 2, …, 19, 20}). The simplex method with the CC target was used to perform six-dimensional positional refinement on these selected helices/strands. Then, the refined helices/strands were used for voxel size determination. For each *S*_*n*_, the original voxel size of the working map (expressed as *v*_*ori*_) was multiplied by a factor (expressed as *m*) from 0.9 to 1.1 with 0.001 intervals (*m*∈ {0.9, 0.901, …, 1.099, 1.1}). Notably, when the voxel size was altered, the entire map expanded or shrank along the *x*, *y* and *z* axes. The coordinates of every grid point (except the origin) on the map then mismatched relative to the already placed templates *S*_*n*_. Therefore, the distance of this additional offset was complemented to adjust the position of *S*_*n*_ after multiplication. Subsequently, simplex positional refinement was performed on the *S*_*n*_ and then followed by CC calculation. For each particularly varying voxel size value *v*_*ori*_ * *m*, the CC between the refined template *S*_*n*_ and the working cryoEM map was calculated and expressed as *CC*_*mn*_. All the *CC*_*mn*_ values were accumulated across the top 20 helices/strands as: $\sum _{n}C{C}_{mn}$. The maximum value of accumulated *CC*_*mn*_ values determined the corresponding voxel size adjustment scale, as:

3 $${{v}}_{{new}}={{v}}_{{ori}}\ast \mathop{\text{arg}\,\max }\limits_{m}\{\sum _{n}C{C}_{mn}\}$$

For each cryoEM map, helix and strand adjustment ratios were balanced to calculate an overall novel voxel size by weighted-averaging the voxel sizes from these two sources. The weight was dependent on the number of “good” helices and strands (CC > 0.3) found in the map.

### Map Simulation

At the Cα stage of model building, the scaling factor between the reference and working maps was adjusted according to their power spectra. The Guinier plots of the working and reference structures were calculated separately. The natural logarithm of the structure factor (expressed as *lnF*) of the reference structure was interpolated to the *lnF* of the working structure at the resolution beyond 10 Å. At the low-resolution region (<10 Å) the reference map was interpolated to the nearest 10 Å resolution to prevent overweighting. Once the scale was applied to the reference map, it was also low-pass filtered to the same resolution as that of the working map to generate the simulated reference map. The resulting simulated map was then used to accumulate the log-likelihood target function for the subsequent Cα identification and possible sequence assignment if the sequence was available.

## Results

### Model Building

**Figure 2:**
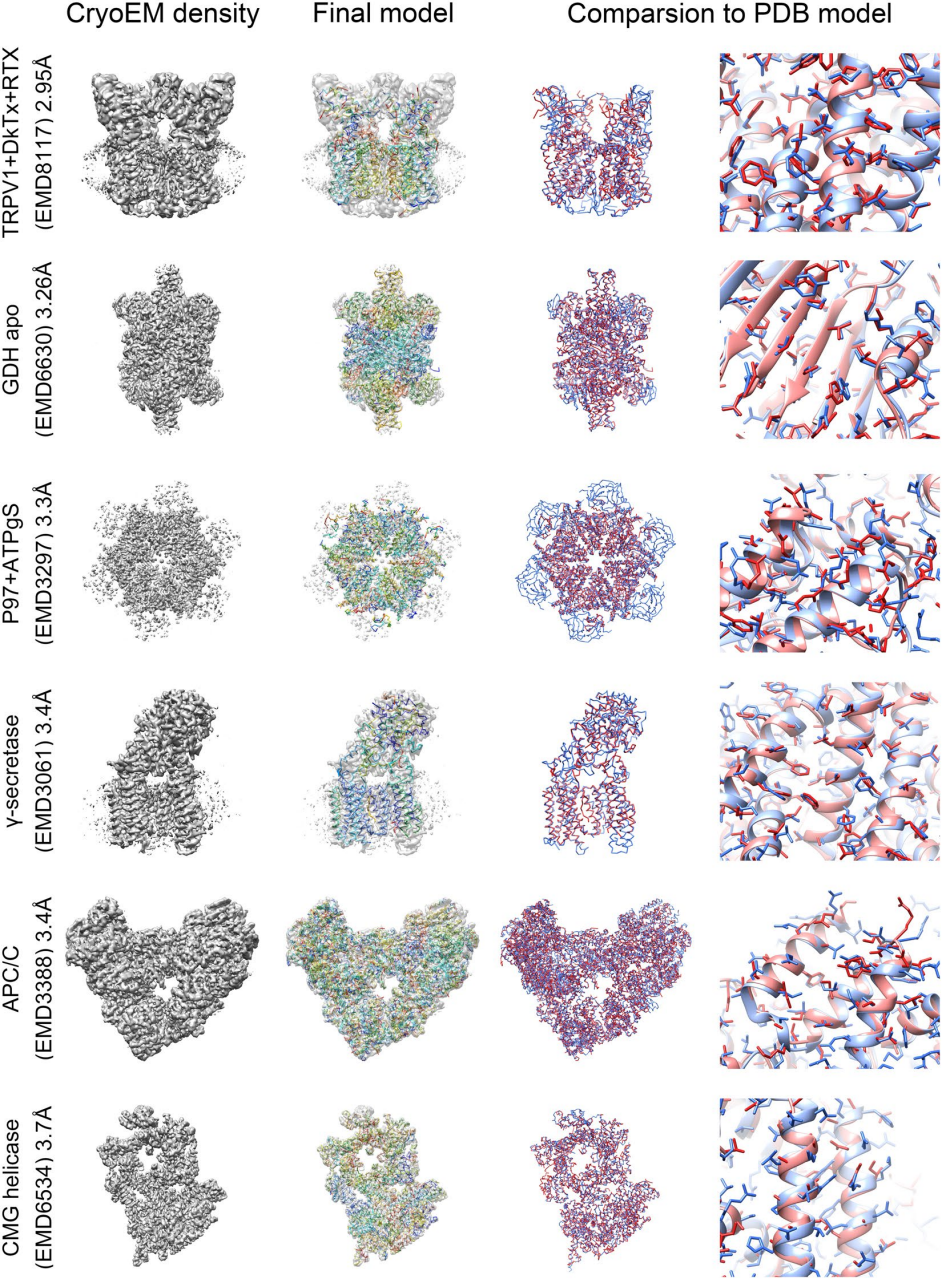
Model-building results from EMBuilder and comparison with PDB models. The cryoEM density map is presented in gray surface in the first column with a threshold from EMDB. Models built with EMBuilder are shown in the second column as cartoon representation. The overall comparison of the models built with EMBuilder (red ribbon) and the PDB models (blue ribbon) is presented in the third column. The side chain assignments of the models built with EMBuilder (red stick) and the PDB model (blue stick) are given in the fourth column.

The entire model-building procedure in EMBuilder was performed on benchmark test data sets containing 6 cryoEM maps from the EMDataBank^25^ (EMDB) (Fig. 2), *i.e*. EMD8117^28^ (M.W. 300 kDa, 2.95 Å), EMD6630^29^ (M.W. 336 kDa, 3.26 Å), EMD3297^30^ (M.W. 540 kDa, 3.3 Å), EMD3061^2^ (M.W. 170 kDa, 3.4 Å), EMD3388^31^ (M.W. 1.2 MDa, 3.4 Å) and EMD6534^32^ (M.W. 700 kDa, 3.7 Å). The parameters used in the model building were as the followings: reference map: EMDB8194; resolution: reported in EMDB; number of residues to be built: estimated on the basis of molecular mass; and helix/strand templates: generated from EMD2984 after low-pass filtering to 2.5 Å.

**Figure 3:**
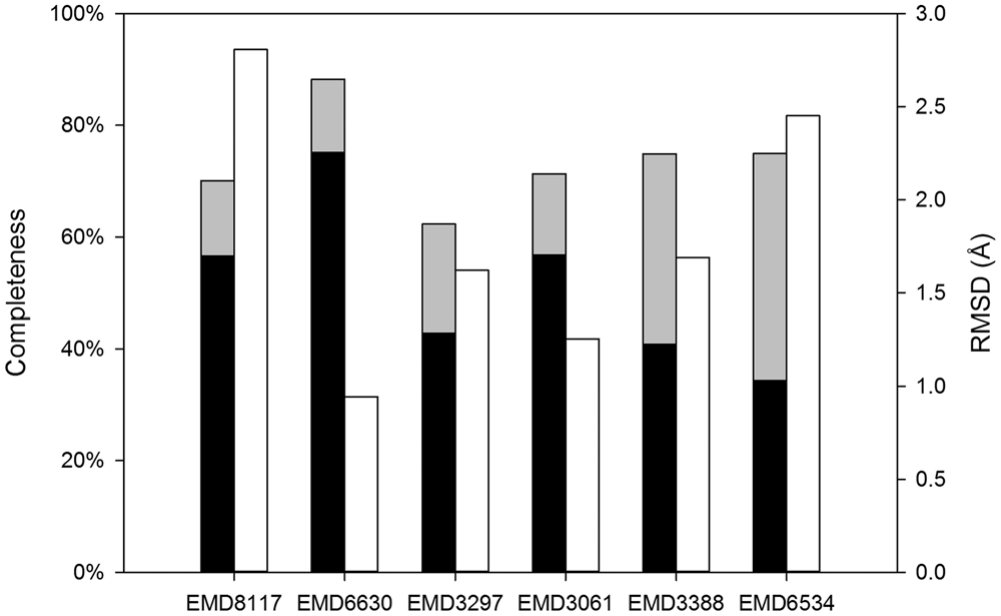
Completeness and RMSDs of the models built by EMBuilder from the test data sets. The shaded bars and white bars represent the completeness and RMSD, respectively. In the shaded bars, black and gray represent the percentages of Cα built between 1 Å and 2 Å and within 1 Å, respectively.

We calculated the completeness, Cα RMSD (Fig. 3) and accuracy of side chain assignment (Supplementary Table S1) to measure the quality of the model-building results. Cα RMSD values were calculated by measuring the distance between each Cα in the model and the nearest Cα in the final structure deposited in the PDB. The RMSDs of the auto-built model ranged from 0.9 Å to 2.8 Å for our test data sets. On average, ~51% of the Cαs were built within 1 Å of the structure deposited in the PDB, and ~73% of the Cαs were built within 2 Å. Although the main chain, especially Cα positions could be identified to the reasonable quality in the map, the difficulty of assigning side chains for a cryoEM map is varying across the whole map because of the uneven resolution distribution of the map. If the side-chain density is good enough for visualization in a local area of a cryoEM map, the assignment will be successful by using our simulation and side-chain assignment method (Supplementary Table S1). The running times required for model building for EMD8117, EMD6630, EMD3297, EMD3061, EMD3388 and EMD6534 were 1.5 h, 3.3 h, 5.5 h, 1 h, 23.5 h and 5.5 h, respectively, on a 2.6-GHz central processing unit (CPU).

In the central core regions of these six maps, the features of each residue are unambiguous, such that the LLK target function can identify the spatial orientations of the main and side chains. Therefore EMBuilder produced low-RMSD models (*e.g*. the transmembrane domains of TRPV1 and γ-secretase). Nonetheless in the peripheral region, which usually only has medium resolution because of radiation damage or particle misalignment or domain flexibility, the recognition of residues by the target function is difficult. Some error may occur even in main chain tracing, especially in loop building. The RMSD of the model in this region is ~0.5 Å greater than that at core region. Therefore, EMBuilder is suitable for generating high-resolution cryoEM models. Moreover, EMBuilder can build models for large cryoEM maps. The PDB model of EMD3388 comprises ~7300 residues. In our model, 3282 Cαs were built within 1 Å of the Cαs in the PDB model, and 6009 Cαs were built within 2 Å. These results indicate that substantial model-building time can be saved using EMBuilder compared with manual building.

### Voxel Size Refinement

**Figure 4:**
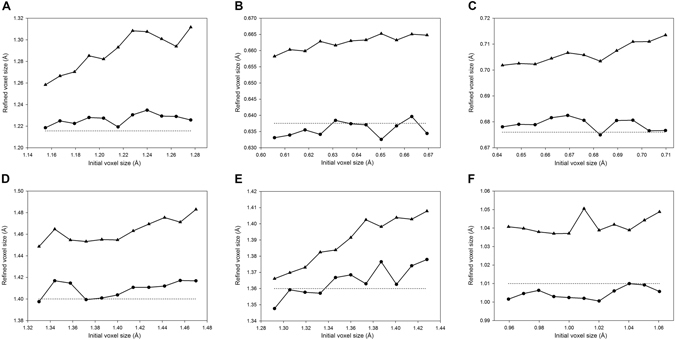
Results of voxel size refinement. The test data sets are: EMD8117 (**A**), EMD6630 (**B**), EMD3297 (**C**), EMD3061 (**D**), EMD3388 (**E**) and EMD6534 (**F**). RESOLVE template and our template (2.5 Å) were used in the refinement and are depicted as triangles and circles, respectively. The dotted line represents the correct voxel size of the map (from EMDB).

The voxel sizes of cryoEM maps may be inaccurate, and the error can be as great as 5%^17^. Therefore, the voxel size must be assessed or corrected before model building. The voxel size refinement subroutine in EMBuilder was designed to perform this task. We manually introduced voxel size error to evaluate the accuracy of our voxel size refinement subroutine. We assumed that all the maps deposited in the EMDB had the correct voxel sizes. The error was introduced before refinement by multiplying the voxel size by a factor ranging from 0.95 to 1.05 in the intervals of 0.01, which corresponded to errors of ±5%. We used 4 templates to evaluate the accuracy: one from RESOLVE^20^, and 3 computed from a cryoEM map after low-pass filtering to 2.5 Å, 3.0 Å and 3.5 Å (described in Methods). The refined voxel size was compared with the correct voxel size to validate the accuracy (Fig. 4). The detailed data of voxel size refinement can be found as Supplementary Figure S1.

Our voxel size refinement subroutine reduced the voxel size error to 0.59% on average. The best refinement result reduced the voxel size error to 0.37%, a value acceptable for model building. Our template with low-pass filtering to 2.5 Å yielded the best accuracy for voxel size refinement among the templates tested (data not shown). Therefore, it was used as the default template in EMBuilder. Moreover, the template from RESOLVE clearly had relatively high error (~4%) in voxel size refinement compared with those of our templates (Fig. 4), thus suggesting that the features of cryoEM maps differ from those of the maps used in crystallography. Therefore, the parameters and algorithms used for crystallography may not generate optimal results when they are directly applied to cryoEM maps.

### Map Simulation

**Figure 5:**
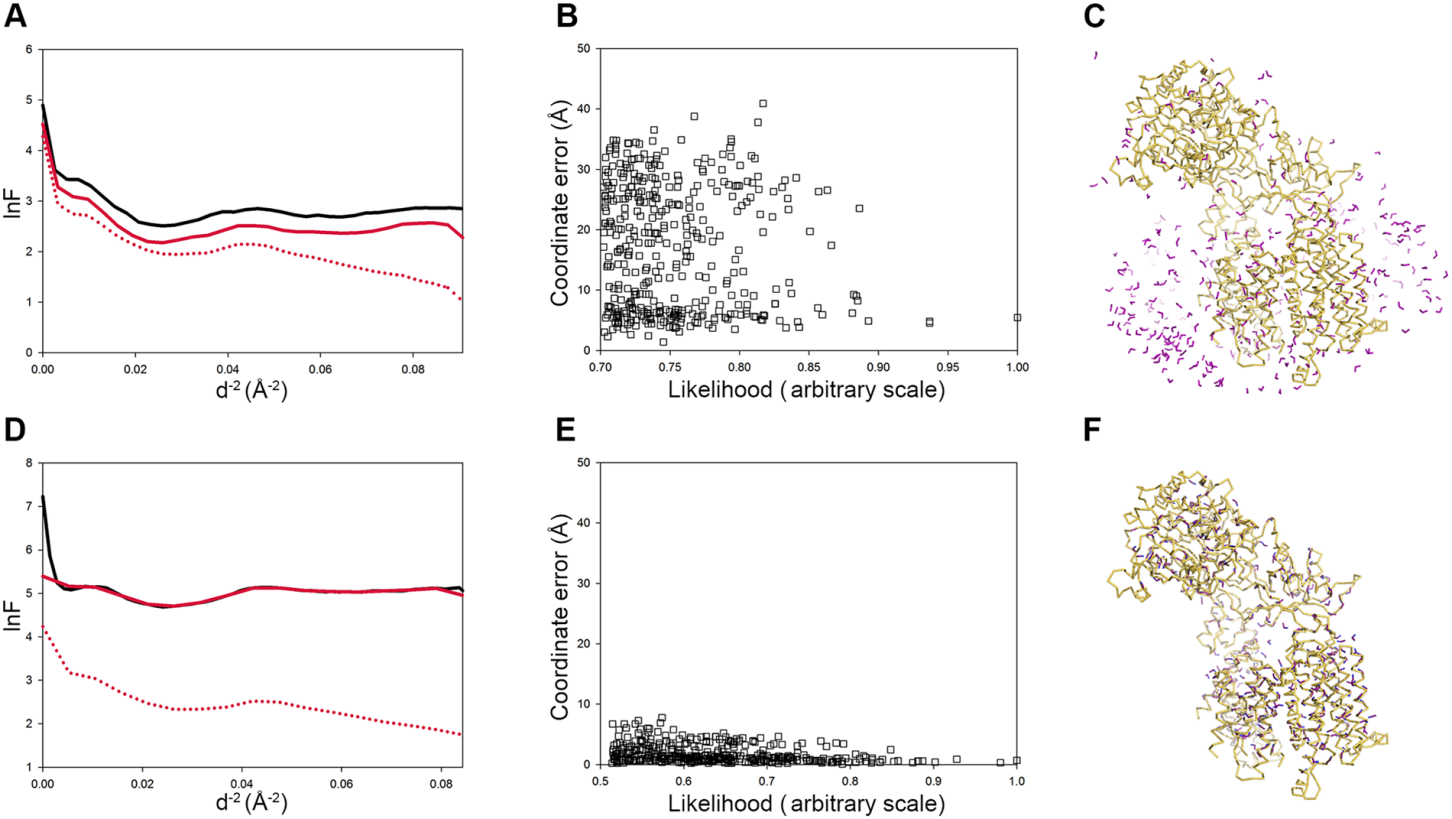
Accuracy evaluation of the map simulations of Buccaneer and EMBuilder. The *lnF vs d* ^−2^ plots of the working map, original reference map and simulated reference map are shown as a solid black line, dotted red line and solid red line, respectively (panels A and D). The Cα distances between the structures deposited in PDB and the Cα finding are presented in panels B and E, respectively. The structures deposited in PDB and the Cα finding are shown as yellow ribbons and purple sticks, respectively (panels C and F).

During the Cα stage of model building, the generation of the LLK target function requires a reference map and a working map on the same scale. The purpose of the map simulation subroutine is to adjust the scale of the reference map to that of the working map. The map simulation and Cα finding were performed with EMBuilder and Buccaneer^11^ on the test data sets to specifically evaluate the effectiveness of our map simulation subroutine. Buccaneer, which was designed specifically for model building in crystallography, may also be used for cryoEM map model building, but its power is suboptimal. The working map was EMD3061, and the reference map was EMD8194. We calculated the Guinier plot (*lnF vs. d* ^−2^) of the reference and the working structures (Fig. 5A,D) and Cα distance between the structures deposited in the PDB and the Cα finding (Fig. 5B,E).

The map simulation method used by EMBuilder was effective for cryoEM maps and offered better results than Buccaneer. The percentage of Cαs placed by EMBuilder within 2 Å of those in the structures deposited in the PDB was 70.8%. The Cα finding in Buccaneer was influenced by the density of the detergent in the map because of inappropriate map simulation (Fig. 5C). In contrast, most of the Cαs identified by EMBuilder were located on the backbone of the protein (Fig. 5F), thus suggesting that the algorithm used for crystallography may not perform optimally when applied to cryoEM maps.

## Discussion

We developed EMBuilder, which uses a template-matching method for model building of cryoEM maps. This program is capable of correcting the voxel size error and building atomic models for high-resolution (<3.5 Å) cryoEM maps. EMBuilder uses two stages to build an atomic model. The positions of helices and strands are identified and refined in seven dimensions in the secondary structure stage. In the subsequent Cα stage the reference cryoEM map is adjusted to the same scale as that of the working map to generate the LLK target functions of the main chain and side chain. Then, the Cαs are extended into main chain fragments according to the density map. The side chains are assigned on the basis of the assembled main chain fragments. The time required for model building is trivial enough compared with manual building. For a 300-kDa protein, 2 to 3 h was required for EMBuilder to build a model with ~70% completeness, a time considerably less than that required in manual building. Additionally, the time required to build a very large cryoEM density map (~1 MDa) was acceptable (~24 h) with a single thread.

A previous study has reported that the voxel size error in cryoEM maps can be as great as ~5%^17^. Under normal circumstances, the voxel size error of a cryoEM map may be ~2%. Additionally, the voxel size error can accumulate across several residues during residue extension, thus severely affecting the fitting between the model and map. Therefore, voxel size correction is an essential procedure before model building. However, it is difficult to correct the voxel size of a map manually when the corresponding model is unavailable. Our solution involves 1) identifying secondary structure elements (SSEs) in the map, 2) correcting the voxel size of the SSEs, and 3) using the results of the corrected SSEs’ voxel sizes to determine the overall voxel size. Thus, the method corrects the SSE voxel size by calculating the CC with the pre-computed template and adjusts it until the CC is maximized. After obtaining the voxel sizes of the SSEs, the 20 helices and strands with the highest CCs are used to determine the overall voxel size. By using our test data sets, we demonstrated that this process is robust. The entire refinement procedure required only ~2 minutes in most cases. Thus, our voxel size refinement subroutine provides an easy method of correcting the voxel size of a cryoEM map.

We also found that the choice of templates significantly influences the accuracy of voxel size refinement. The template derived from the crystallography density map yielded ~4% error in voxel size refinement. By using our templates, we found that the voxel size refinement subroutine reduced the voxel size error to 0.59% on average; this value is acceptable for model building. One possible reason for this difference is that the density distribution and pattern of noise of crystallography maps and cryoEM maps are not similar.

In a crystallographic model-building program, such as Buccaneer, the map simulation method is used to adjust the scaling factors of two maps according to the Wilson statistics^11^. However, we found that the Buccaneer simulation method produced minimal effect in cryoEM maps, thus resulting in the misidentification of Cαs, possibly because of the higher noise level in the cryoEM maps. We have tested several methods to equivalently scale the cryoEM maps and found that linear interpolation of *lnF vs d* ^−2^ between the reference and working maps was rapid and produced acceptable accuracy.

Resolution typically varies widely across cryoEM maps. Therefore, a different model-building strategy should be used if a map contains both high-resolution and medium-resolution regions. For medium-resolution and ambiguous regions, more external information, such as secondary structure prediction, can be added to aid in determining the molecular topology. In addition, the map can be segmented by resolution. In these sub-maps, different algorithms and templates can be used to build local models. In regions of lower resolution, automatic docking between the pre-computed homology model and the cryoEM map can be performed. Thus, the resolution range of input map for EMBuilder can be extended to a lower level. Moreover, a mask of the asymmetric unit might be created for a homo-multimer structure. Then, the speed of model building can be accelerated by building the model into only one asymmetric unit.

In conclusion, we present a model-building program—EMBuilder—for high-resolution cryoEM maps. This program is based on a template-matching method that uses pre-computed templates to correct voxel sizes and build atomic models. EMBuilder is an effective program that can help researchers build cryoEM structure models rapidly and easily.

## Electronic supplementary material

Supplementary Information
