# Supplementary material for: EMBuilder: A Template Matching-based Automatic Model-building Program for High-resolution Cryo-Electron Microscopy Maps
Source: Sci Rep. 2017 Jun 1;7:2664. doi: 10.1038/s41598-017-02725-w (PMC5453991; doi:10.1038/s41598-017-02725-w)
Supplement: Supplementary file 1 — Supplementary Information [file 41598_2017_2725_MOESM1_ESM.pdf]

EMBuilder: A Template Matching-based Automatic Model-building Program for High-resolution Cryo-Electron Microscopy Maps

Niyun Zhou, Hongwei Wang, and Jiawei Wang

Supplementary Table S1. Accuracy of the side chain assignment of EMBuilder on the test data sets.

EMDB entry of test data sets	Resolution (Å)	Successful assigned residues	Total assigned residues	Sidechain assignment accuracy
8117	2.95	776	968	80.17%
6630	3.26	1962	2339	83.88%
3297	3.3	1029	2643	38.93%
3061	3.4	323	457	70.68%
3388	3.4	594	1804	32.93%
6534	3.7	34	409	8.31%

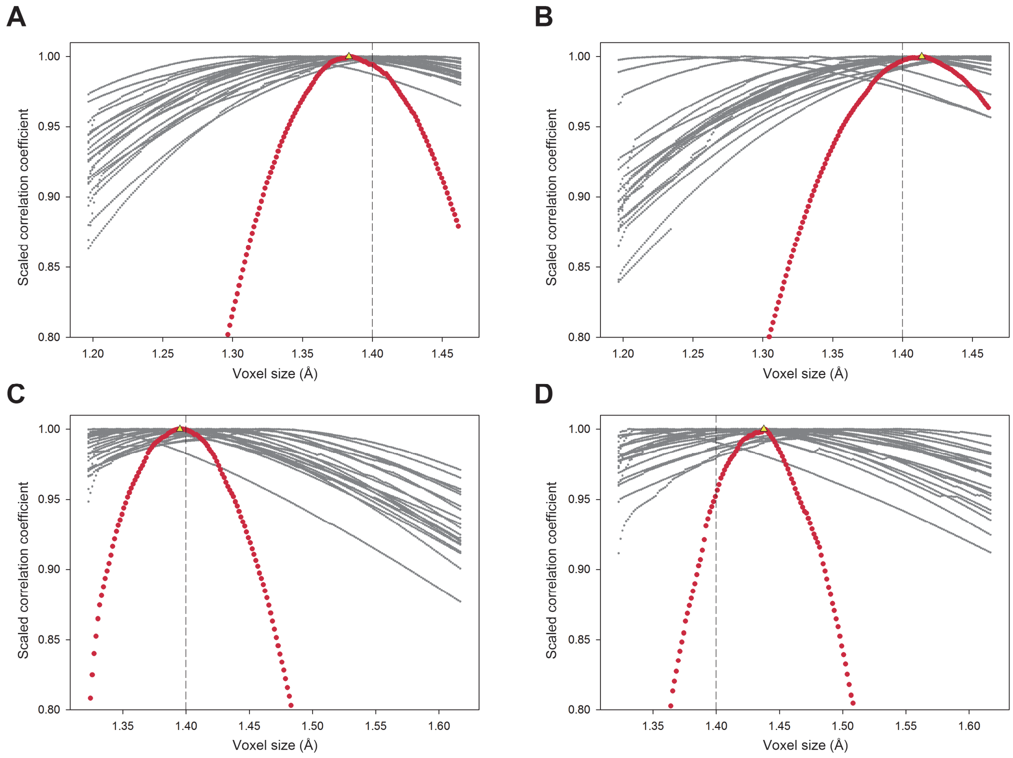

Supplementary Figure S1. Voxel size refinement on the cryoEM map of γ-secretase (EMDB entry 3061). The initial voxel size is set to -5% (panel A, B) and +5% (panel C, D), respectively. The helix template (panel A, C) and strand template (panel B, D) are used for the refinement. Each grey line represents the scaled CCs of a single helix or strand template. The red line represents the scaled sum of all CCs (grey lines) and the yellow triangle represents the maximum value of red line (refined voxel size of helices or strands). The dashed vertical line represents the deposited voxel size of the map.
